# Deep Neural Networks as a Computational Model for Human Shape Sensitivity

**DOI:** 10.1371/journal.pcbi.1004896

**Published:** 2016-04-28

**Authors:** Jonas Kubilius, Stefania Bracci, Hans P. Op de Beeck

**Affiliations:** Brain and Cognition, University of Leuven (KU Leuven), Leuven, Belgium; University of Tübingen and Max Planck Institute for Biologial Cybernetics, GERMANY

## Abstract

Theories of object recognition agree that shape is of primordial importance, but there is no consensus about how shape might be represented, and so far attempts to implement a model of shape perception that would work with realistic stimuli have largely failed. Recent studies suggest that state-of-the-art convolutional ‘deep’ neural networks (DNNs) capture important aspects of human object perception. We hypothesized that these successes might be partially related to a human-like representation of object shape. Here we demonstrate that sensitivity for shape features, characteristic to human and primate vision, emerges in DNNs when trained for generic object recognition from natural photographs. We show that these models explain human shape judgments for several benchmark behavioral and neural stimulus sets on which earlier models mostly failed. In particular, although never explicitly trained for such stimuli, DNNs develop acute sensitivity to minute variations in shape and to non-accidental properties that have long been implicated to form the basis for object recognition. Even more strikingly, when tested with a challenging stimulus set in which shape and category membership are dissociated, the most complex model architectures capture human shape sensitivity as well as some aspects of the category structure that emerges from human judgments. As a whole, these results indicate that convolutional neural networks not only learn physically correct representations of object categories but also develop perceptually accurate representational spaces of shapes. An even more complete model of human object representations might be in sight by training deep architectures for multiple tasks, which is so characteristic in human development.

## Author Summary

Shape plays an important role in object recognition. Despite years of research, no models of vision could account for shape understanding as found in human vision of natural images. Given recent successes of deep neural networks (DNNs) in object recognition, we hypothesized that DNNs might in fact learn to capture perceptually salient shape dimensions. Using a variety of stimulus sets, we demonstrate here that the output layers of several DNNs develop representations that relate closely to human perceptual shape judgments. Surprisingly, such sensitivity to shape develops in these models even though they were never explicitly trained for shape processing. Moreover, we show that these models also represent categorical object similarity that follows human semantic judgments, albeit to a lesser extent. Taken together, our results bring forward the exciting idea that DNNs capture not only objective dimensions of stimuli, such as their category, but also their subjective, or perceptual, aspects, such as shape and semantic similarity as judged by humans.

## Introduction

Understanding how the human visual system processes visual information involves building models that would account for human-level performance on a multitude of tasks. For years, despite the best efforts, computational understanding of even the simplest everyday tasks such as object and scene recognition have been limited to toy datasets and poor model performances. For instance, hierarchical architecture HMAX [1], once known as “the standard model” of vision [2], worked successfully on a stimulus set of paper clips and could account for some rapid categorization tasks [3] but failed to capture shape and object representations once tested more directly against representations in the visual cortex (e.g., [4–6]).

Recently, however, deep neural networks (DNNs) brought a tremendous excitement and hope to multiple fields of research. For the first time, a dramatic increase in performance has been observed on object and scene categorization tasks [7,8], quickly reaching performance levels rivaling humans [9]. More specifically in the context of object recognition, stimulus representations developed by the deep nets have been shown to account for neural recordings in monkey inferior temporal cortex and functional magnetic resonance imaging data throughout the human ventral visual pathway (e.g., [6,10,11]), suggesting that some fundamental processes, shared across different hardware, have been captured by deep nets.

The stimulus sets on which DNNs have been tested in these previous studies allow the inference that there is a general correspondence between the representations developed within DNNs and important aspects of human object representations at the neural level. However, these stimulus sets were not designed to elucidate specific aspects of human representations. In particular, a long tradition in human psychophysics and primate physiology has pointed towards the processing of shape features as the underlying mechanism behind human object recognition (e.g., [12–15]). Cognitive as well as computational models of object recognition have mainly focused upon the hierarchical processing of shape (e.g., [1,16,17]). There are historical and remaining controversies about the exact nature of these shape representations, such as about the degree of viewpoint invariance and the role of structural information in the higher levels of representation (e.g., [18,19]). Still, all models agree on the central importance of a hierarchical processing of shape. For this reason we hypothesized that the general correspondence between DNNs representations and human object representations might be related to a human-like sensitivity for shape properties in the DNNs.

Here we put this hypothesis to the test through a few benchmark stimulus sets, which have highlighted particular aspects of human shape perception in the past. We first demonstrate that convolutional neural networks (convnets), the most common kind of DNN models in image processing, can recognize objects based upon shape also when all other cues are removed, as humans can. Moreover, we show that despite being trained solely for object categorization, higher layers of convnets develop a surprising sensitivity for shape that closely follows human perceptual shape judgments. When we dissociate shape from category membership, then abstract categorical information is available to a limited extent in these networks, suggesting that a full model of shape and category perception might require richer training regimes for convnets.

## Results

### Experiment 1: Recognition from shape

**Fig 1:**
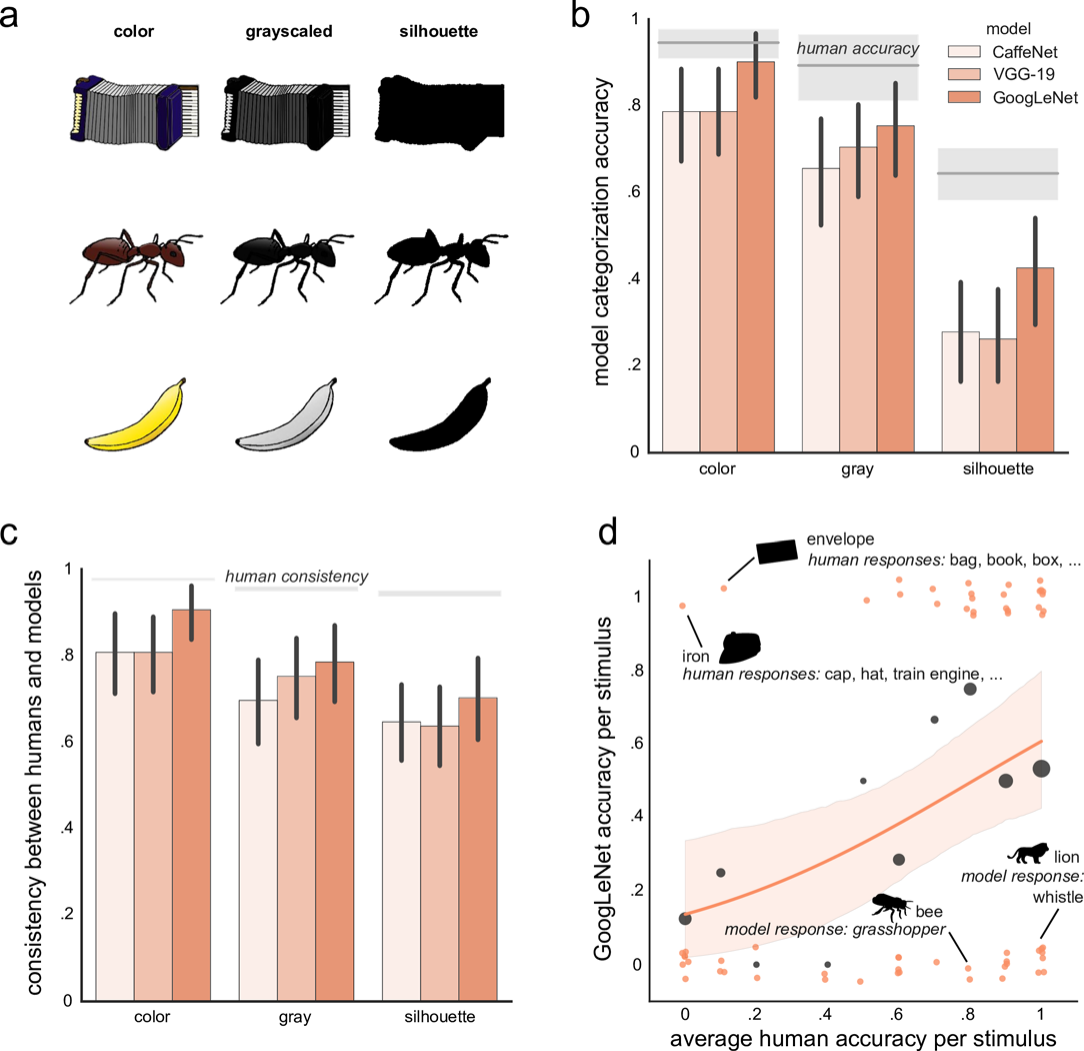
Object categorization. (a) Examples of stimuli from the modified Snodgrass and Vanderwart stimulus set [21]. Stimulus images courtesy of Michael J. Tarr, Center for the Neural Basis of Cognition and Department of Psychology, Carnegie Mellon University, http://www.tarrlab.org/. (b) Human (*n* = 10 for each variant of the stimulus set) and convnet (CaffeNet, VGG-19, GoogLeNet) accuracy in naming objects. For each stimulus set variant, mean human performance is indicated by a gray horizontal line, with the gray surrounding band depicting 95% bootstrapped confidence intervals. Error bars on model performance also depict 95% bootstrapped confidence intervals. (c) A consistency between human and convnet naming of objects. A consistency of .5 means that about half of responses (whether correct or not) we consistent between a model and an average of humans. Error bars indicate 95% bootstrapped confidence intervals. Gray bands indicate estimated ceiling performance based on between-human consistency. (d) Correlation with human performance on the silhouette stimulus set. The x-axis depicts an average human accuracy for a particular silhouette [15] and the y-axis depicts GoogLeNet performance on the same silhouette (either correct (value 1.0) or incorrect (value 0.0)). Model’s performance is jittered on the x- and y-axis for better visibility. Dark gray bubbles indicate average model’s performance for 11 bins of human performance (i.e., 0–5%, 5–15%, 15–25%, etc.) with the size of each bubble reflecting the number of data points per bin. The orange line shows the logistic regression fit with a 95% bootstrapped confidence interval (light orange shaded). The slope of the logistic regression is reliably different from zero.

If convnets are indeed extracting perceptually relevant shape dimensions, they should be able to utilize shape for object recognition. This ability should extend to specific stimulus formats that highlight shape and do not include many other cues, such as silhouettes. The models have been trained for object recognition with natural images, how would they perform when all non-shape cues are removed? In order to systematically evaluate how convnet recognition performance depends on the amount of available shape and non-shape (e.g., color or texture) information, we employed the colorized version of the Snodgrass and Vanderwart stimulus set of common everyday objects [20,21]. This stimulus set consists of 260 line drawings of common objects that are easily recognizable to human observers and has been used extensively in a large number of studies (Google Scholar citations: over 4000 to [20]; over 500 to [21]). In our experiments, we used a subset of this stimulus set (see Methods), consisting of 61 objects (Fig 1A). Three variants of the stimulus set were used: original color images, greyscale images, and silhouettes.

First, we asked 30 human observers (10 per variant of the stimulus set) to choose a name of each object, presented for 100 ms, from a list of 657 options, corresponding to the actual of these objects and their synonyms as defined by observers in [20]. Consistent with previous studies [15,21], participants were nearly perfect in naming color objects, slightly worse for grayscale objects, and considerably worse for silhouettes (Fig 1B, gray bands). Moreover, we found that participants were very consistent in their responses (Fig 1C, gray bands).

We then presented three convnets with the stimuli and asked them to produce a single best guess of what might be depicted in the image. A correct answer was counted if the label exactly matched the actual label. We found that all deep nets exhibited a robust categorization performance on the original color stimulus set, reaching about 80–90% accuracy (Fig 1B, with the best model (GoogLeNet) reaching human level of performance. Given that the models have not been trained at all on abstract line drawings, we found it an impressive demonstration of convnet feature generalization.

As textural cues were gradually removed, convnets still performed reasonably well. In particular, switching to grayscale decreased the performance by about 15%, whereas a further decrease by 30% occurred when inner gradients were removed altogether (silhouette condition). In other words, even when an object is defined solely by its shape, convnets maintain a robust and highly above-chance performance. Notably, a similar pattern of performance was observed when humans were asked to categorize these objects, suggesting that models are responding similarly to humans but are overall less accurate (irrespective of stimulus variant).

To investigate the consistency between human and model responses in more detail, we computed a squared Euclidean distance between the average human accuracy and a model accuracy, and normalized it to the range [0, 1], such that a consistency of .5 means that a model responded correctly where a human responded correctly and made a mistake where a human made a mistake about half of the time (Fig 1C; see Methods for reasoning behind this choice of consistency). Overall, the consistency was substantial and nearly reached between-human consistency for color objects for our best model (GoogLeNet). To visualize the amount of consistency, we depicted The best model’s (GoogLeNet) performance on silhouettes against human performance (Fig 1D). The performances are well correlated as indicated by the slope of the logistic regression being reliably above zero (Fig 1D; z-test on GoogLeNet: *z* = 2.549, *p* = .011; CaffeNet: *z* = 2.393, *p* = .017; VGG-19: *z* = 2.323, *p* = .020). Furthermore, we computed consistency between models and found that for each variant of the stimulus set, the models appear to respond similarly and commit similar mistakes (the between-model consistency is about .8 for each pairwise comparison), indicating that the models learn similar features.

Fig 1D also shows that the models sometimes outperformed humans, seemingly in those situations where a model could take an advantage of a limited search space (e.g., it is much easier to say there is an iron when you do not know about hats). Overall, however, despite the moderate yet successful performance on silhouettes, it is obvious from Fig 1D that there are quite some stimuli on which the models fail but which are recognized perfectly by human observers. Common mistakes could be divided into two groups: (i) similar shape (*grasshopper* instead of *bee*), and (ii) completely wrong answers where the reason behind model’s response is not so obvious (*whistle* instead of *lion*). We think that the former scenario further supports the idea that models base their decisions primarily on shape and are not easily distracted by the lack of other features. In either case, the errors might be remedied by model exposure to cartoons and drawings. Moreover, we do not think that these discrepancies might be primarily due to the lack of recurrent processes in these models since we tried to minimize influences of possible recurrent processes during human categorization by presenting stimuli for 100 ms to human observers. It is also possible that better naturalistic training sets in general are necessary where objects would be decoupled from background. For instance, lions always appear in savannahs, so models might be putting too much weight on savannah’s features for detecting a lion, which would be a poor strategy in the case of this stimulus set. Nonetheless, even in the absence of such training, convnets generalize well to such unrealistic stimuli, demonstrating that they genuinely learn some abstract shape representations.

### Experiment 2: Physical vs. perceived shape

In Experiment 2, we wanted to understand whether convolutional neural networks develop representations that capture the shape dimensions that dominate perception, the so-called “perceived” shape dimensions, rather than the physical (pixel-based) form. In most available stimulus sets these two dimensions are naturally correlated because the physical form and the perceived shape are nearly or completely identical. In order to disentangle the relative contributions of each of these dimensions, we needed stimulus sets where a great care was taken to design perceptual dimensions that would differ from physical dimensions.

#### Experiment 2a

**Fig 2:**
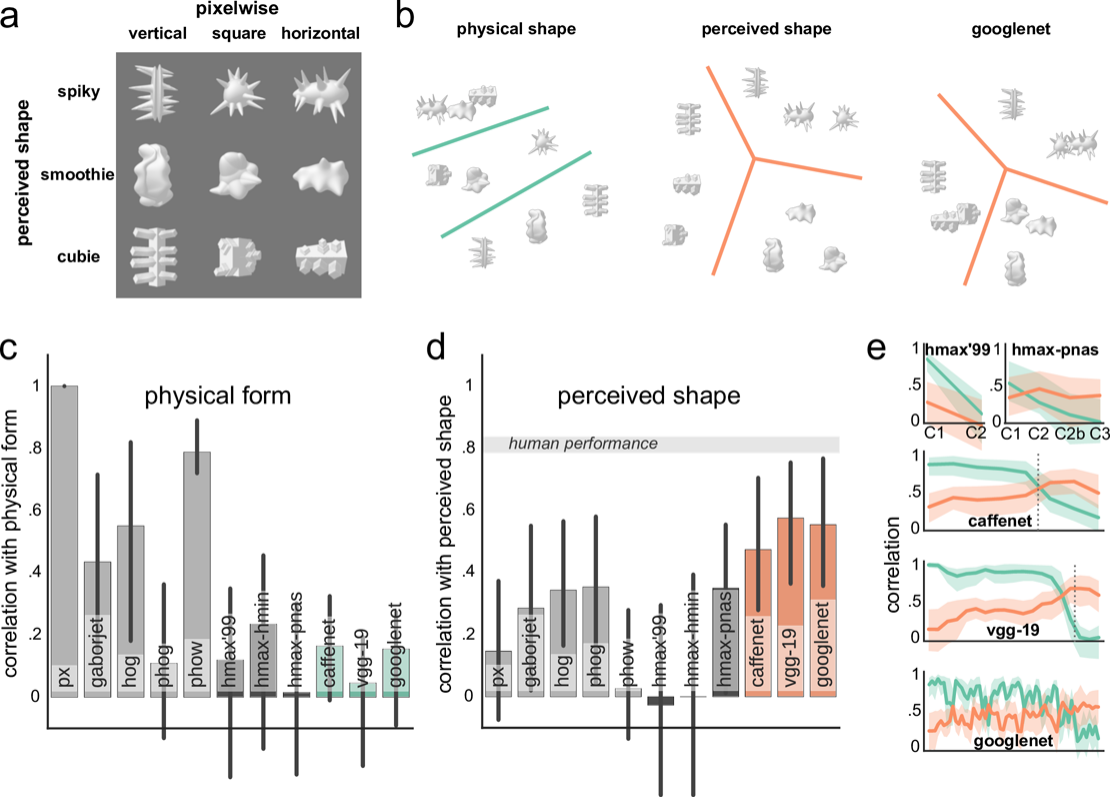
Model preference for shape. (a) Stimulus set with physical and perceived shape dimensions manipulated orthogonally [5]. (b) Multidimensional scaling plots for the dissimilarity matrices of physical form, perceived shape, and the GoogLeNet outputs (at the top layer). The separation between shapes based on their perceived rather than physical similarity is evident in the GoogLeNet outputs (for visualization purposes, indicated by the lines separating the three clusters). (c) A correlation between model outputs and the physical form similarity of stimuli. Most shallow models are capturing physical similarity reasonably well, whereas HMAX and deep models are largely less representative of the physical similarity. (d) A correlation between model outputs and the perceived shape similarity of stimuli. Here, in contrast, deep models show a tendency of capturing perceived shape better than shallow and HMAX models. Gray band indicates estimated ceiling correlation based on human performance. (e) Correlation with physical (green) and perceived (orange) shape similarity across the layers of HMAX models and convnets. A preference for the perceived shape emerges in the upper layers. Vertical dotted lines indicate where fully-connected layers start. In all plots, error bars (or bands) indicate the 95% bootstrapped confidence intervals.

First, we used a stimulus set where the physical and perceptual dimensions were specifically designed to be orthogonal. Building on their earlier stimulus set [22], Op de Beeck and colleagues [5] created a stimulus set of nine novel shapes (Fig 2A) that can be characterized either in terms of their overall shape envelope / aspect ratio (vertical, square, horizontal), which we refer to as a physical form, or their perceptual features (spiky, smoothie, cubie), which we refer to as a perceived shape since humans base their shape judgments on these features, as explained below. They then computed the physical form dissimilarity matrix by taking the difference in the pixels, whereas the perceptual shape dissimilarity matrix was obtained in a behavioral experiment by asking participants to judge shape similarity ([5]; see Methods for details). They reported that participants typically grouped these stimuli based on the perceived shape and not shape envelope (Fig 2B shows the multidimensional scaling plot of these dissimilarity matrices), and also provided neural evidence for such representations in the higher shape-selective visual area known as the lateral occipital complex (LOC). The most common hierarchical model of object recognition available at that time, the original HMAX [1], did not capture perceived shape with this stimulus set.

In order to investigate the relative importance of the physical form and perceived shape for various computer vision models, we used this stimulus set to compute the outputs of five shallow (single layer) models (pixelwise, GaborJet, HOG, PHOG, and PHOW), three versions of HMAX of varying complexity (HMAX ‘99, HMAX-HMIN, HMAX-PNAS), and three deep convnets (CaffeNet, VGG-19, and GoogLeNet; see Methods for details). Next, we computed the dissimilarity between each pair of the nine stimuli, resulting in a 9x9 dissimilarity matrix, and we applied multidimensional scaling on these matrices. Fig 2B shows the two-dimensional arrangement derived from multidimensional scaling for CaffeNet. This procedure revealed that the representations of these stimuli in the output of deep models tended to cluster based on their perceived rather than physical similarity, comparable to human judgments and neural representations in the higher visual areas in human cortex [5].

To quantify how well the computed stimulus dissimilarities overall corresponded to the physical form or the perceived shape dissimilarity, we correlated physical form and perceived shape dissimilarity matrices with model’s output. We found that shallow models were mostly better at capturing physical dissimilarity than the output layers of deep models (Fig 2C; bootstrapped related samples one-tailed significance test for shallow vs. HMAX and for shallow vs. deep: p < .001), whereas perceived shape was better captured by most deep models (Fig 2D; bootstrapped related samples one-tailed significance test for deep vs. HMAX: *p* = .010; deep vs. shallow: *p* = .002). (But note that, obviously, early layers of deep nets typically can reflect physical dissimilarities too.) Correlation layer-by-layer revealed that preference for shape gradually increased throughout the layers of all convnets, whereas physical similarity abruptly decreased at their upper layers (Fig 2E).

#### Experiment 2b

**Fig 3:**
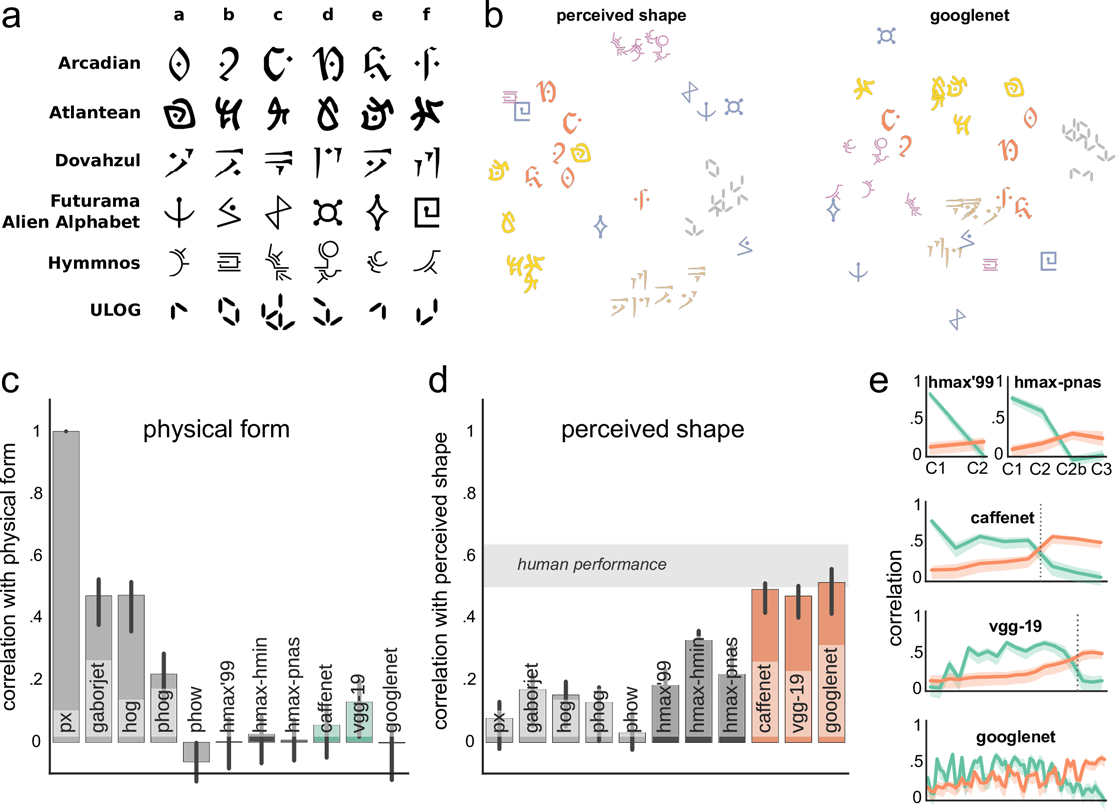
Model preference for shape. (a) Stimulus set of six letters from six constructed fonts. (b) Multidimensional scaling plots for the dissimilarity matrices of shape judgments by humans and the GoogLeNet outputs (at the top layer). Notice how humans and GoogLeNet are good at clustering the ULOG font but both struggle with Futurama. (c-d) A correlation between model outputs and physical form similarity (c) and perceived shape similarity (d) of stimuli. Whereas shallow models only capture physical shape, deep models capture perceived shape significantly better than shallow and HMAX models. Gray band indicates the estimated ceiling correlation based on human performance. (e) Correlation with physical (green) and perceived (orange) shape similarity across the layers of HMAX models and convnets. Vertical dotted lines indicate where fully-connected layers start. A preference for the perceived shape emerges in the upper layers. In all plots, error bars (or bands) indicate the 95% bootstrapped confidence intervals.

So far, we found that deeper networks generally reflect perceive shape similarity better than shallower ones. Is this effect specific to the two dimensions used in [5] or does it reflect a broader tendency for deeper nets to develop perceptually-relevant representations? In Experiment 2b, we constructed a new stimulus set where perceptually relevant dimensions were no longer explicitly defined. In particular, it was composed of six letters from six novel font families (Fig 3A). Even though the letters were unrecognizable (e.g., a letter ‘a’ in these fonts looked nothing like a typical ‘a’) and varied substantially within a font family, implicitly the letters in each font shared the same style (size was matched across fonts as much as possible). It should be noted though that even for human observers detecting these font families is not straightforward (note mistakes in Fig 3B).

As seen in Fig 3C, deep models are sensitive to differences between fonts, with the letters presented in the same font tending to be clustered in the same part of the representational space. To quantify the similarity of model representations to human judgments, as before, we asked participants to rate the similarity between all these letters, and correlated their judgments with model outputs (Fig 3D). We found that deep models captured perceived similarity of letters significantly better than shallow models (bootstrapped related samples significance test, one-tailed: *p* < .001), whereas shallow models correlated significantly better with physical form (bootstrapped related samples significance test, one-tailed: *p* < .001), consistent with our hypothesis that the formation of perceptually-relevant representational spaces is a general property of convnets. In fact, convnets captured all explainable variance in human data, demonstrating that in certain scenarios convnets are already sufficiently robust.

Moreover, we observed that convnets exhibited significantly stronger correlations with behavioral similarity than HMAX family of models (bootstrapped related samples significance test, one-tailed: *p* < .001). This tendency was already visible in Exp. 2a, but did not reach statistical significance yet. With this larger stimulus set that contains less pronounced perceptual dimensions, the limitations of HMAX models in shape processing and their relevance in understanding human perception became prominent, even for the most complex version HMAX-PNAS. These results corroborate with earlier observations that HMAX family of models are not sufficient for explaining categorical data dimensions [4,6].

### Experiment 3: Non-accidental properties

**Fig 4:**
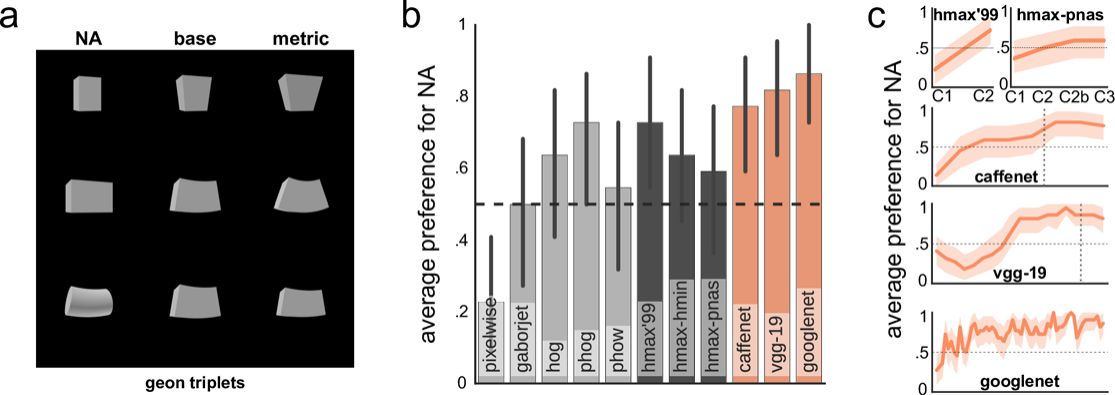
(a) Examples of geons [24]. In order to measure model’s sensitivity to changes in non-accidental properties, model’s output is computed for a particular stimulus (middle column) and compared to the output when another variant of the same kind of stimulus is presented (right column) and when a non-accidental change in the stimulus is introduced (left column) that is physically (in the metric space) just as far from the base as the metric variant. We used 22 such triplets in total. (b) Model performance on discriminating between stimuli. For each triplet, model’s output is counted as accurate if the non-accidental variant is more dissimilar from the base stimulus than the metric variant is from the base. Chance level (50%) is indicated by a dashed line. (c) HMAX and convnet performance on the task at different layers. Non-accidental stimuli appear to be closer to the base in the early layers, which is consistent with a conservative design of the stimuli [24]. Best performance is observed at the upper layers of convnets with a slight dip at the output layer. Vertical dotted lines indicate where fully-connected layers start. In both plots, error bars (or bands) depict 95% bootstrapped confidence intervals.

In 1987, Biederman put forward the Recognition-by-Components (RBC) theory [16] that proposed that objects recognition might be based on shape properties known as non-accidental. Under natural viewing conditions, many object’s properties are changing, depending on lighting, clutter, viewpoint and so on. In order to recognize objects robustly, Biederman proposed that the visual system might utilize those properties that remain largely invariant under possible natural variations. In particular, Biederman focused on those properties of object shape that remain unchanged when the three-dimensional shape of an object is projected to the two-dimensional surface on the eye’s retina, such as curved versus straight object axis, parallel versus converging edges, and so on [23]. Importantly, RBC theory predicts that observers should notice a change in a non-accidental property more readily than an equivalent change in a metric property. Consider, for example, geons shown in Fig 4A, top row. Both the non-accidental and the metric variant differ by the same amount from the base geon (as measured by some linear metric, such a pixelwise or GaborJet difference), yet the non-accidental one appears more distinct to us.

Over years, Biederman and others consistently found such preference to hold in a large number of studies across species [25–28], age groups [29–31], non-urban cultures [32], and even in the selectivity of inferior temporal neurons in monkeys [24,33]. This idea of invariants has also been shown to play an important role in scene categorization [34] and famously penetrated computer vision literature when David Lowe developed his SIFT (Scale-Invariant Feature Transform) descriptor that attempted to capture invariant features in an image [35].

Thus, the sensitivity for non-accidental properties presents an important and well-tested line of research where the physical size of differences between shapes is dissociated from the effect of specific shape differences on perception. We tested the sensitivity for non-accidental properties using a previously developed stimulus set of geon triplets where the metric variant is as distinct or, typically, even more distinct from the base than the non-accidental variant as measured in the metric (physical) space. Nevertheless, humans and other species report perceiving non-accidental shapes as more dissimilar from the base than the metric ones, presenting us with a perfect test case where, similar to Exp. 2, physical shape similarity is different from the perceived one.

We evaluated model performance on this set of 22 geons (Fig 4A) that have been used previously in behavioral [31,32,36] and neurophysiological studies. A model’s response was counted as accurate if the response to a non-accidental stimulus was more dissimilar from the base than the metric one.

We found that all deep but not shallow or HMAX models (except for HMAX’99) showed a higher than chance performance (Fig 4B) with performance typically improving gradually throughout the architecture (Fig 4C; bootstrapped related samples significance test for deep vs. shallow, one-tailed: *p* < .001; deep vs. HMAX: *p* = .011). Moreover, deeper networks tended to perform slightly better than shallower ones, in certain layers even achieving perfect performance. Overall, there was not any clear pattern in mistakes across convnets, except for a tendency towards mistakes in the main axis curvature, that is, convnets did not seem to treat straight versus curved edges as very distinct. In contrast, humans consistently show a robust sensitivity to changes in the main axis curvature [31,36]. Note that humans are also not perfect at detecting NAPs as reported by [36]. Thus, we do not go further into these differences because the RBC theory and most previous behavioral and neural studies only address a general preference for NAP changes, and hence do not provide a systematic framework for interpreting the presence or absence of such preference for specific NAPs.

### Experiment 4: The role of category when dissociated from shape

In the first three experiments, we demonstrated convnet sensitivity to shape properties. However, these convnets have been explicitly trained to optimize not for shape but rather category, that is, to provide a correct semantic label. Apparently, categorization is aided by developing sensitivity to shape. But is there anything beyond sensitivity to shape then that convnets develop? In other words, to what extent do these networks develop semantic representations similar to human categorical representations over and above mere shape information?

**Fig 5:**
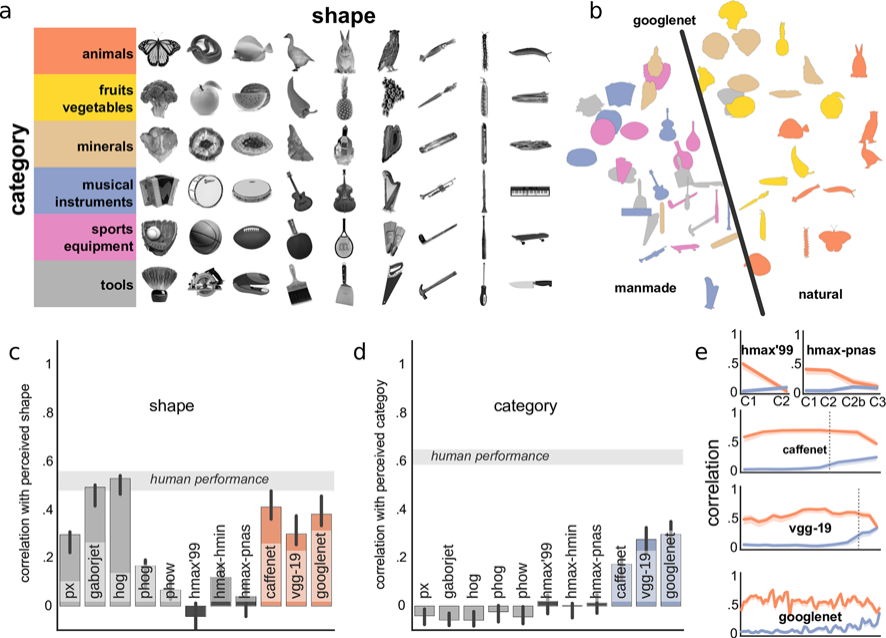
Categorical representations in convnets. (a) Stimulus set from [37] with shape and category information largely orthogonal. (Adapted with permission from [37].) (b) Multidimensional scaling plot representing object dissimilarity in the output layer of GoogLeNet. The black line indicates a clear separation between natural (brown, orange, yellow) and man-made (blue, gray, pink) objects. (c-d) A correlation between model representations and human shape (c) and category (d) judgments. Gray band indicates estimated ceiling correlation based on human performance. (e) A correlation with human shape (orange) and category (blue) judgments across the layers of HMAX models and convnets. Vertical dotted lines indicate where fully-connected layers start. In all plots error bars (or bands) represent 95% bootstrapped confidence intervals.

Typically, object shape and semantic properties are correlated, such that objects from the same category (e.g., fruits) share some shape properties as well (all have smooth roundish shape) that may distinguish them from another category (e.g., cars that have more corners), making it difficult to investigate the relative contributions of these two dimensions. To overcome these limitations, Bracci and Op de Beeck [37] recently designed a new stimulus set, comprised of 54 photographs of objects, where shape and category dimensions are orthogonal to each other as much as possible (Fig 5A). In particular, objects from six categories have been matched in such a way that any one exemplar from a particular category would have a very similar shape to an exemplar from another category. Thus, the dissociation between shape and category is more prominent and can be meaningfully measured by asking participants to judge similarity between these objects based either on their shape or on their category. By correlating the resulting dissimilarity matrices to human neural data, Bracci and Op de Beeck [37]found that perceived shape and semantic category are represented in parallel in the visual cortex.

We employed this stimulus set to explore how categorical information is represented by convnets. As before, participants were asked to judge similarity among stimuli based either on their shape or on their category. Note that even for categorical judgments, participants were asked to rate categorical similarity rather than divide stimulus set into six categories, resulting in idiosyncratic categorical judgments and consistency between humans not reaching ceiling.

First, we found that convnets represented shape fairly well, correlating with perceptual human shape judgments between .3 and .4, nearly reaching the human performance limit (Fig 5C and 5D). Unlike before, the effect was not specific to deep models but was also observed in HMAX and even shallow models. This observation is expected because, unlike in previous experiments, in this stimulus set physical form and perceived shape are well correlated. Instead, the purpose of this stimulus set was to investigate to what extent semantic human category judgments are captured by convnets, since here category is dissociated from shape. We found that all deep but not shallow or HMAX models captured at least some semantic structure in our stimuli (Fig 5D and 5E; bootstrapped related samples significance test for deep vs. shallow and deep vs. HMAX: *p* < .001), indicating that representations in convnets contain both shape and category information. Similar to Exp. 1, comparable correlations were observed even when the models were provided only with silhouettes of the objects (no texture), indicating that such categorical decisions appear to rely mainly on the shape contour and not internal features.

The abundance of categorical information in convnet outputs is most strikingly illustrated in Fig 5B where a multidimensional scaling plot depicts overall stimulus similarity. A nearly perfect separation between natural and manmade objects is apparent. Note that less than a half of these objects (23 out of 54) were known to GoogLeNet, but even completely unfamiliar objects are nonetheless correctly situated. This is quite surprising given that convnets were never trained to find associations between different categories. In other words, there is no explicit reason why a convnet should learn to represent guitars and flutes similarly (the category of “musical instruments” is not known to the model). We speculate that these associations might be learned implicitly, since during training objects of the same superordinate category (“musical instruments”) might co-occur in images. Further tests would be necessary to establish the extent of such implicit learning in convnets.

Despite its significance, the correlation with categorical judgments was much weaker than with shape, even after we restricted stimuli to the 23 objects in the ImageNet, meaning that the learned representations in convnets are largely based on shape and not category. In other words, categorical information is not as dominant in convnets as in humans, in agreement with [6] where deep nets were shown to account for categorical representations in humans only when categorical training was introduced on top of the outputs of convnets. (See also Discussion where we talk about the availability of information in models.)

## Discussion

Here we demonstrated that convolutional neural networks are not only superior in object recognition but also reflect perceptually relevant shape dimensions. In particular, convnets demonstrated robust similarities with human shape sensitivities in three demanding stimulus sets: (i) object recognition based solely on shape (Exp. 1), (ii) correlations with perceived rather than physical shape dimensions (Exp. 2), and (iii) sensitivity to non-accidental shape properties (Exp. 3). Notably, these shape-based representations emerged without convnets being trained for shape processing or recognition and without any explicit knowledge of our stimuli.

Furthermore, we demonstrated that convnets also develop abstract, or superordinate, category representations, but to a much smaller extent than shape (Exp. 4). In particular, we found that objects belonging to the same superordinate category (e.g., bananas and oranges belong to fruits) are represented more similarly than objects from different superordinate categories (e.g., bananas and violins).

These results expand the growing literature that convnets reflect human visual processing [10,11]. More specifically, the correspondence between object representations in primates and convnets has been investigated in the context of object recognition. Our study adds the new information that the powerful object recognition performance of convnets is related to a human-like sensitivity for shape and, to a lesser extent, perceptually-adequate semantic spaces of the objects they learn.

We emphasize that the reported sensitivity to shape reflects the representational spaces learned in convnets, as opposed to the information that is in principle available in them. Both approaches are useful and valid for evaluating how good a particular model is in processing visual information, but they provide different kinds of information. “Available information” means that a linear classifier, such as a Support Vector Machine, can learn (with supervision) to correctly classify stimuli into predefined categories. Thus, if an above-chance classification can be performed from a model’s output, it means that a model is making this the information necessary to perform a task explicit (object manifolds become untangled, as described in [38]). For instance, object categorization based on their pixel values does not work but after a series of transformations in convnets, categorical information becomes available. A better model for a particular task is then the one that has task-relevant information explicitly available.

While this approach provides a valuable information about model’s behavior, it does not directly provide evidence if it is a good model of human visual perception. In other words, the fact that a model is categorizing objects very well does not imply that it represents these categories similarly to humans. An explicit evaluation how well a model explains human data needs to be performed. One approach is to use model outputs to predict neural data, as used in [6,10]. However, in this case model outputs are combined in a supervised fashion, so this approach also resembles the “available information” approach in that we use external knowledge to investigate the amount of information available in the model outputs.

Another approach is to compare the representational spaces in models and humans, as proposed in [39] and used in this study. By computing the similarity of stimulus representations in models and humans we can understand if models implicitly develop representations that match human representations. Critically, we are not asking if a model contains necessary information that could be used for a particular task. Rather, we are asking if a model is already representing this information in a similar way independent of a task. To illustrate this difference, consider, for instance, stimuli in Exp. 2a. Even a very simple shallow model, such as GaborJet, can learn to classify these nine stimuli into three perceptual categories (spikies, smoothies, and cubies) correctly because these dimensions are very simple and are easily accessible. Nonetheless, we found that representations in such simple models do not match human perception. Even though one could decode all the necessary information in stimuli from Exp. 2a to perform classification into perceptual categories, this kind of information is not made dominant in shallow models.

Again, both approaches are valid and meaningful, but it is important to be explicit about the implications that they bring. We emphasize that, despite only being trained on a task that has a clear correct answer to it (i.e., object recognition), convnets also develop representations that reflect subjective judgments of human observers. In our opinion, this difference is critical to the success of deep nets. Observe that in the typical convnet training, a particular image can have only a cat or a dog, or a car, or something else, always with a correct answer for any given image, and deep nets have been shown to learn this correspondence very well. However, this is very different from what humans typically do. The human visual system is particularly adept at making stable judgments about the environment when there is no clear or single answer to a given problem. For example, most individuals would report seeing a house rather that a stack of bricks even though both answers are technically correct. As a recent blunder of Google Photos app that labelled black people as gorillas [40] illustrates, a machine that is incapable of arriving to perceptually congruent decisions might be unacceptable in social settings where such common sense is expected. Although more data would clearly help, it is hard if not impossible to have training data for every possible situation. Uncertainty is bound to occur in natural scenarios (e.g., due to the lack of training samples or poor visibility), so a more error-prone strategy is making sure that machines are learning perceptually relevant dimensions that will generalize properly to unfamiliar settings. Such machines can become better than humans, but critically when they are wrong, their mistakes will be human-like. We therefore expect that further advancement of computer vision and artificial intelligence at large are critically dependent not only on improvement in benchmark performance but also in matching human perceptual judgments.

Our data also provide insights how convnets relate to the information processing across the visual cortex. First, we observed that early layers of convnets tended to relate to the physical stimulus dimensions, consistent with the known properties and models of early visual cortex [41]. Second, the output layer of convnets related to the perceived shape properties. Earlier human neuroimaging studies and monkey electrophysiological recordings revealed that these perceptual shape representations are implemented in human occipitotemporal cortex and in monkey inferotemporal cortex. Specifically, fMRI experiments with the stimulus set used in our Exp. 2a have shown that the shape-selective lateral occipital area might be mostly involved in this shape processing [5]. Moreover, the geon stimulus set used in Exp. 3 also showed disproportionate sensitivity to non-accidental properties in monkey physiological recordings [24]. Finally, the stimulus set used in Exp. 4 showed a co-existence of shape and category information in human visual cortex and the dominance of categorical representations in the most anterior parts of it [37]. Taken together, these results suggest that the shape representations in output layers of convnets relate to shape processing in higher visual areas in primates and their behavioral responses.

However, note that it is not necessarily the output layer which provides the best fit with shape representations in the primate brain. Given that the output layer is directly optimized to produce a correct category label rather than to represent shapes, it is possible that earlier layers are in fact better at capturing shape dimensions. Our results appear to be broadly consistent with this notion. However, these differences between layers seem to be small and the best intermediate layer is not consistent across experiments. Moreover, shape itself is a hierarchical concept that can be understood at multiple scales of analysis (e.g., local, global) and at multiple layers of abstraction [42], so it may not be possible to pinpoint an exact locus of shape representations neither in convnets nor in the human visual system. Rather, different dimensions of shape features might be distributed across multiple areas.

### Causes for shape sensitivity in convnets

Our results suggest that a human-like sensitivity to shape features is a quite common property shared by different convnets, at least of the type that we tested. However, the three convnets were also very similar, since all of them very trained on the same dataset and used the same training procedure. Which convnet properties are important in developing such shape sensitivity?

One critical piece of information is offer by the comparison to HMAX models. Despite a similar architecture, in most experiments we observed that overall HMAX models failed to capture shape sensitivity to the same extent as convnets. The most obvious difference lies in the depth of the architecture. There are at most four layers in HMAX models but at least eight layers in the simplest of our convnets, CaffeNet. However, HMAX’99 (that has two layers) did not seem to perform consistently worse than HMAX-PNAS (that has four layers). Another important difference is the lack of supervision during training. As has been demonstrated before with object categorization [6], unsupervised training does not seem to be sufficiently robust, at least the way it is implemented in HMAX.

Another hint that supervision might be the critical component in learning universal shape dictionaries comes from comparing our results to the outputs obtained via the Hierarchical Modular Optimization (HMO) that was recently reported to correspond well to primate neural responses [10]. For Exps. 2a and 4, where we could obtain the outputs of the HMO layer that corresponds best to monkey neural data, we found largely similar pattern of results, despite differences in depth, training procedure, and training dataset. The only clear similarity between the tested convnets and HMO was supervised learning.

Finally, part of convnet power might also be attributed to the fully-connected layers. Both in CaffeNet and VGG-19, the critical preference for perceived shape emerges at the fully-connected layers. In GoogLeNet, the preference to perceptual dimensions is typically the strongest at the last layer that is also fully-connected, though earlier layers that are not fully-connected also exhibit a robust preference for perceived shape.

Other parameters, such as the naturalness of the training dataset or the task that convnet is optimized for, might also contribute to the representations that convnets develop. In short, the tests and the models that we have included in the present paper provide a general answer to our hypotheses about shape representations in convnets, but there are many specific questions about the role of individual variables that remain to be answered.

### Relation to theories of shape processing

In the literature, at least two theoretical approaches to shape processing have played an important role: image-based theories [19], which capitalize on processing image features without an explicit encoding of the relation between them, and structure-based theories [18], which emphasize the role of explicit structural relations in shape processing. Our results do not necessarily provide support for particular theories of shape processing. Of course, in their spirit convnets are closer to image-based theories since there is no explicit shape representation computed. On the other hand, in Exp. 3 we also found that convnets were sensitive to non-accidental properties even without ever being trained to use these properties. While in principle HMAX architectures can also develop sensitivity to non-accidental properties when a temporal association rule is introduced [43], the fact that such sensitivity automatically emerges in convnets when training for object categorization provides indirect support that non-accidental properties are diagnostic in defining object categories, as proposed by the RBC theory [16].

Of course, a mere sensitivity to non-accidental properties does not imply that convnets must actually utilize the object recognition scheme proposed by the RBC theory [16]. For instance, according to this theory, objects are subdivided into sets of shape primitives, known as geons, and recognized based on which geons compose that particular object, referred to as a “structural description” of the object. Finding an increased sensitivity for non-accidental properties does not necessarily imply that all these other assertions of the RBC theory are correct, and it does not by itself settle the controversy between image-based and structure-based models of object recognition.

### Are convnets *the* model of human visual processing?

While we demonstrate an unprecedented match between convnet representations and human shape perception, our experiments only capture a tiny fraction of the rich repertoire of human shape processing. It is clear from Exp. 1 that despite a strong performance, convnets remain about 20% worse than human observers at object recognition on silhouettes. Given that convnets are already very deep and were trained exhaustively, it may be a sign that in order to bridge this gap, convnets need additional layers dedicated to developing more explicit structural representations.

Another, more fundamental limitation is their feedforward architecture. Whereas humans are thought to be able to perform many object and scene recognition tasks in a feedforward manner [44–46], they are certainly not limited to feedforward processing and in many scenarios will benefit from recurrent processing [47]. The role of such recurrent processes has been particularly debated in understanding perceptual organization, where the visual system is actively organizing the incoming information into larger entities [48]. For instance, monkey neurophysiology revealed that figure-ground segmentation benefits both from feedforward and feedback processes [49], and many models of segmentation utilize recurrent loops (for an in-depth discussion, see [50]). In contrast, despite their superior object categorization abilities, vanilla convnets show rather poor object localization results, with the top-performing model (GoogLeNet) in the ImageNet Large Scale Visual Recognition Challenge 2014 scoring 93.3% on a single object categorization task, yet localizing that object with only 73.6% accuracy [51].

In other words, we showed that convnets sensitivity to shape that reflects human judgments once the object itself can be easily extracted from the image. However, as soon as segmentation and other perceptual organization processes become more complicated, humans but not convnets can benefit from recurrent connections. Thus, recurrent neural networks, which incorporate the feedforward complexity of the tested convnets, might provide an even better fit to human perception than purely feedforward convnets.

Finally, we have also argued in [50] that feedforward architectures such as convnets might be lacking critical mechanisms that could contribute to the initial image segmentation. In our view, high performance at object recognition and shape processing tasks should not be taken as evidence that the “convolution-non-linearity-pooling” stack at the heart of convnets is necessarily the right or the full solution of feedforward visual processing yet. Small modifications to this architecture, such as adding feature map correlations [52,53] or performing VLAD [54] or Fisher Vector [55] pooling already provides convnets with the ability to segment input images and represent textures and artistic style, all of which might be the part of feedforward computations in human visual cortex.

Taken together, we demonstrated that convolutional neural networks trained for multiclass object categorization implicitly learn representations of shape that reflect human shape perception. Moreover, we showed that convnets also develop abstract semantic spaces independent of shape representations that provide a good, albeit weaker, match to human categorical judgments. Overall, our results provide an important demonstration that convnets are not limited to only extracting objective information from the visual inputs (such as object category) but can also represent the subjective aspects of visual information in accordance to human judgments. In other words, our work suggests that convnets might be a good candidate model for understanding various perceptual qualities of visual information.

## Methods

### Ethics statement

Studies reported here were approved by the Social and Societal Ethic Committee at KU Leuven (Exp. 1 and 2b) and the Massachusetts Institute of Technology’s Committee on the Use of Humans as Experimental Subjects (Exp. 1).

### Materials

Almost all simulations were run with Python using the *psychopy-ext* package [56] that provides several simple shallow models and bindings to the Caffe library [57] and to several popular computer vision models (PHOG, PHOW, HMAX-HMIN, and HMAX-PNAS), written in MATLAB/C. For online data collection, we used *mturkutils*, a Python interface package to Amazon Mechanical Turk. For data collection in in Exp. 2b, we used similarity rating interface in MATLAB from [58].

For data analysis, we used several popular free and open source Python packages, including *numpy*, *scipy*, *scikits-learn*, *scikits-image* [59], *pandas*, *seaborn*, *statsmodels*, and *NLTK* [60]. The code and stimulus sets for all of our simulations are available publicly at https://osf.io/jf42a, except in the cases when the stimulus set is already available online (links to these stimulus sets are provided in the repository and in the text) or subject to copyright restrictions (stimulus set for Exp. 4). For a maximal reproducibility, all results reported in this manuscript can be generated with a single command: python run.py report—bootstrap.

### Stimulus sets

All stimuli were scaled to 256×256 px size. Convnets further downsampled these images to their own predefined image sizes (typically around 224×224 px). Stimuli pixel intensities were rescaled to the range between 0 and 1, where 0 corresponds to a black pixel and 1 corresponds to a white pixel, and, for deep models, the mean of the ImageNet training set was subtracted. No further processing was done.

#### Snodgrass and Vanderwart stimulus set of common objects [20]

This is a popular set of 260 common everyday objects (one exemplar per category). The complete stimulus set is available at http://wiki.cnbc.cmu.edu/images/SVLO.zip. In our experiments, we used its updated version that is freely available and contains images in [21]. Moreover, since not all object categories in this stimulus set were contained in the training set of convnets, we selected a subset of categories for our experiments. First, for each category, we determined their WordNet synsets [61], so that we could directly relate them to the ImageNet categories used for convnet training. We selected only those categories that exactly matched the ones in the ImageNet training set. While we could have attempted to include more categories by allowing more specific terms (e.g., *apple* could have been included since *Granny Smith* is in the ImageNet training set), it is not clear whether such an inclusion would be valid. For instance, it is possible that only a green apple would be labeled as an apple whereas a red one would be more often labeled as a peach or a cherry simply because the models were trained on green apples only. Thus, we opted for a conservative set of 61 categories.

Starting from the color images, we produced two further variants of the stimulus set:

- Grayscaled images by converting the original images to 256 shades of gray.
- Silhouettes by filling the entire object with black. Note that this procedure also fills in any cavities inside objects. For example, the silhouette of a donut would appear as a disk. We used this procedure to be consistent with the stimuli used by [15].

Behavioral human responses were collected online using Amazon Mechanical Turk platform. For each variant of the stimulus set, ten participants were recruited to type the names of each object. The full stimulus set of 260 stimuli was used in this task. Each participant completed only one variant of the stimulus set. An image was shown for 100 ms, followed by a gap of 500 ms until a response field appeared. The participants were asked to type the name of the object they saw. To make the task more similar to model evaluation where a model is choosing from a list of available nouns, participants could only type answers from the list of 260 categories, their synonyms (as reported in [20]), and several extra synonyms that appeared to be valid but were not present in[20] (namely, *stroller* and *pram* for *baby carriage*, *doorhandle* for *doorknob*, *fridge* for *refrigerator*, and *bunny* and *hare* for *rabbit*). In total, there were 657 options. Initially, the response field was empty but as participants typed in the first three characters, a list of suggestions appeared, and the participants were forced to choose from them. A correct response was counted if participant’s response either exactly matched the original name or was in the list of synonyms. Note that for several categories some synonyms overlapped.

We requested that only participants fluent in English completed the task, and participants could stop doing the task at any point if they felt it was too difficult for them. However, as there is no simple way to enforce this requirement in Amazon Mechanical Turk, it is possible that some of the reported mistakes resulted from the lack of fluency in English. On the other hand, on average participants performed slightly better than reported in previous well-controlled studies [15,21] despite much shorter stimulus presentation times, though this may also be driven by the differences in naming procedure (free labelling in those studies as compared to a choice task in our case). Moreover, there was a substantial agreement in naming patterns between [15] on silhouettes and our study (correlation = .79, consistency = .94). The study took about half an hour to complete and participants were paid 50 cents for their help.

CaffeNet, VGG-19, and GoogLeNet were provided with the images from these three variants to produce a single best guess for each image. To match this task to human responses, the models could only choose from one of 61 possible answers (that is, responses not in the list of our 61 categories were not permitted). A correct answer was recorded that label matched the original synset. While a smaller search space may give an advantage to models as compared human observers who had a much larger search space (657 concepts), we note that humans (but not models) were also permitted to provide rather generic responses (such as *bird* instead of *peacock*), which should have made the task easier. However, it is possible that human performance would be even better if they were to choose among 61 categories only. (that is, the original synset or one of its synonyms).

#### Op de Beeck et al. stimulus set of spiky, smoothie, cubie [5]

Op de Beeck and colleagues [22] investigated shape representations in human visual cortex using a novel generative stimulus set of three-dimensional shapes, belonging to one of three categories:

- Spikies–shapes with spiky appearance and sharp local features
- Smoothies–shapes defined my smooth surfaces
- Cubies–shapes consisting of largely parallel and orthogonal surfaces

Additionally, several parameters controlling the overall shape properties, such as aspect ratio and symmetry, can be manipulated. For their later study, Op de Beeck and colleagues [5] created a stimulus set of nine classes where the shape envelope (physical form) and stimulus category (or “perceived shape”) were manipulated orthogonally. They presented the 20 exemplars from each class in various sequences and asked eight participants to rate shape similarity of the current shape to the preceding one. These shapes were also used in a separate functional magnetic resonance imaging (fMRI) experiment.

For our experiments, we used the behavioral data and the prototype (average exemplars) from the nine shape classes, as reported in the fMRI experiment. All resources are shared in the code repository of this project.

#### Fonts stimulus set

We compiled a new stimulus set specifically for this paper using the first six letters from six constructed fonts: Arcadian, Atlantean, Dovahzul, Futurama Alien Alphabet, Hymmnos, and ULOG (fonts were downloaded from the internet; the resulting stimuli are provided in the code repository of the project). The specific fonts were chosen arbitrarily. The letters were scaled to be approximately the same size, and presented in black on a white background. We asked eight participants (2 males, 6 right-handed) to arrange these 36 shapes on the screen according to their similarity using the interface from [58]. The resulting distances in this two-dimensional space were projected into a multidimensional dissimilarity matrix using the method proposed by [58]. Similarity judgments took about 50 minutes to complete and participants received course credit for their help.

#### Kayaert et al. geon stimulus set [24]

Kayaert and colleagues [24] created a stimulus set of 22 geon triplets, where each triplet consists of a non-accidental and a metric geon that each differ slightly from the base geon but in opposite directions. Whereas the metric variant appears rather similar to the base stimulus, the non-accidental variant differs from the base stimulus in terms of a particular ‘non-accidental’ feature. Such features remain stable in their two-dimensional retinal projection independent of the viewing angle and have been postulated as a basis of object recognition [16]. For example, a curved stimulus axis remains curved from nearly all viewing angles (except for several very specific cases, known as ‘accidental views’, where it is seen straight). In contrast, a right angle does not remain right in a two-dimensional projection, and thus is not a non-accidental feature. Importantly, despite the fact that perceptually non-accidental variants appear more dissimilar from the base that the metric ones, when measured by their pixelwise or gaborjet difference, that is, using some simple linear metric, non-accidental variants are in fact more similar to the base geons than the metric ones.

This stimulus set has been used in several subsequent studies [31,32,36] and is freely available online at http://geon.usc.edu/~ori/.

#### Bracci and Op de Beeck stimulus set [37]

In order to study how the visual system represents shape and category and whether the two dimensions are processed by the same visual areas, [37] created a stimulus set where these two dimensions are orthogonal. In particular, for each stimulus in a particular category there is another stimulus in another category that has been carefully matched in terms of its shape. The stimulus set consists of six categories (minerals, animals, fruits/vegetables, music, sport equipment, and tools) that could be further divided into natural (minerals, animals, fruits/vegetables) and manmade (music, sport equipment, tools). Each category is composed of nine grayscale objects, each with a unique shape within a category, thus the stimulus set could also be divided into nine shape groups.

In a behavioral experiment, [37] asked 16 participants to judge object similarity by dragging stimuli on the screen such that more similar stimuli were closer (multiple object arrangement method; [52]). For shape similarity, participants were asked to arrange the images based on perceived object shape similarity. For semantic category similarity, participants were asked to arrange the images based on the semantic similarity among objects. The resulting distances in this two-dimensional space were projected into a multidimensional dissimilarity matrix using the method proposed by [52], and averaged across participants for correlational analyses in this manuscript.

Due to copyright restrictions, this stimulus set is only available upon request from the authors.

### Models

We used three groups of models: shallow, HMAX, and deep. Shallow models consist of a single layer of processing, and all features are built manually (i.e., there is no training). In contrast, HMAX and deep networks have a hierarchical feedforward architecture and have been trained for object categorization. However, HMAX models are not as deep (up to four layers) and have not been trained very optimally (either by manual feature selection or by imprinting stimulus selectivity), whereas deep nets acquire their features through training by backpropagation, which operates on all the weights in the network.

#### Shallow models

To compare deep net performance to several shallow (single-layer) architectures, we ran our experiments on the following popular models:

- **Pixelwise** model that simply provides the values of all pixels as an output. This model provides a useful baseline for controlling how much low-level information is available in a raw image prior to any processing. In our tests, we used a Python implementation of the pixelwise model, available in the *psychopy_ext* package [56].
- **GaborJet** model [62], a simplistic V1-like model used as a baseline in many studies by Biederman and others (e.g., [31]). This model computes a convolution of an image with a set of Gabor filters of five spatial frequencies and eight orientations, placed on a regular 10x10 grid. In our tests, we used a Python implementation of GaborJet, available in the *psychopy_ext* package [56].
- **Histogram of Oriented Gradient (HOG)** model [63] that produces a histogram of normalized gradients (i.e., orientations) in local image patches. Thus, it computes the distribution of orientations present in the image without caring much where these orientations are located in the image. In our experiments, we used a HOG implementation available from the *scikit-image* Python package [59] with the default parameters of nine gradient orientations and 3x3 image patches of 8x8 px each.
- **Pyramid Histogram of Oriented Gradient (PHOG)** model, introduced by [64] and inspired by the PHOW descriptor [65], that computes the HOG descriptor over several spatial scales and concatenates them. The resulting PHOG descriptor is similar to PHOW but is much simpler (and presumably better) and similar to SIFT but is much denser since features are extracted at all locations. We used a MATLAB implementation available from the authors’ website (http://www.robots.ox.ac.uk/~vgg/research/caltech/phog.html) with four spatial scales (dividing the image into 4, 16, and 64 bins, as well as using the whole image), eight quantization bins, and the range of orientations from 0° to 360°.
- **Pyramid Histogram of Visual Words (PHOW)** model [65] that computes a dense SIFT descriptor (that captures local orientations) over multiple scales [35] and quantizes the computed features across all images using k-means, thus learning a multiscale bag of visual words for that stimulus set. Then feature matching to this dictionary is performed for all those scales in the image, and the resulting histograms are concatenated. We used the authors’ MATLAB implementation (http://web.engr.illinois.edu/~slazebni/research/SpatialPyramid.zip) with default parameters (three spatial scales, corresponding to the whole image and its division into 4 and 16 boxes, and a dictionary of 200 words).

#### HMAX models

HMAX is a family of architectures that consist of a hierarchy of simple and complex layers. Simple layers are built to respond to particular features in the input (e.g., edges, corners), whereas complex layers max pool over outputs of the simple units to introduce invariance to feature transformations (e.g., translation, scale). Over the years, several generations of HMAX models have been proposed:

- **HMAX’99** is the original HMAX architecture proposed by [1]. The first layer, S1, features Gabor filters of four spatial frequency bands and four orientations. Locally maximally responding units are then pooled in layer C1. Next, combinations of these edge filters are produced in layer S2, roughly mimicking corner detection, and a maximum in computed in layer C2. A final View-Tuned Unit (VTU) layer can be introduced to perform template matching to a specific dataset (not used in our experiments). This model is no longer actively used, but remains relevant for a comparison to earlier studies that used it as a benchmark. In our tests, we used a Python port of this model, available in the *psychopy_ext* package [56]. Since convnets usually treat convolution and pooling as a part of a single layer, here (and in other HMAX models) we only report outputs of the C layers and not both S and C. The C layers were chosen because (i) C2 layer but not S2 layer is also the output layer, (ii) HMIN only provides access to the C2 layer, and (iii) HMAX-PNAS by default only reports C layers, so for comparison with previous results C layers are more important. The VTU layer was not used.
- **HMIN** is the minimal implementation of the current HMAX model. It is still composed of the four layers as HMAX’99 but, critically, layer S2 is no longer manually crafted but trained on natural images by imprinting unit responses to these images (see the Supplementary text of [3] for details). We used the model from the authors’ website (http://cbcl.mit.edu/jmutch/hmin/) with the default parameters and the default dictionary of 4075 features. Only data from layer C2 is available.
- **HMAX-PNAS** is the most elaborated version of HMAX that has been shown to perform rapid animal detection is a similar fashion to humans [3]. It is a deeper hierarchy than the original model, composed of nine layers (S1, C1, S2, C2, S2b, C2b, S3, C3, S4), and features bypass routes as well as training of S2, S2b, and S3 layers on natural images, as described for HMIN. We used the model from the authors’ website (http://cbcl.mit.edu/software-datasets/pnas07/index.html) with default parameters, except that all available features were used (C1: 78244, C2: 3124000, C2b: 2000, C3: 2000), whereas the default was selecting 1500 random features for all layers.

#### Deep models

In order to explore the generality of shape processing in convnets, the experiments were conducted using three popular convolutional neural networks (convnets). In a nutshell, convnets are simply multilayer perceptrons with two unique properties: (i) convolutions, such that inputs are convolved with a small kernel, typically around 3×3 px, which means that computations are restricted to local neighborhoods only and that massively reduces the number of computations, and (ii) weight sharing, such that all units (“neurons”) in a particular layer have the same weights, which massively reduces the number of parameters in the model. These simplifications are critical in building models that a manageable, that is, fit in computer memory and can be trained. To increase representational power, each layer contains multiple feature maps (typically in the order of hundreds) each trained to respond to different features in the input.

A layer in a convnet typically is a stack of thee operations: (i) convolution, (ii) non-linearity (usually rectified linear unit (ReLU), and (iii) max and/or average pooling that can be thought of as analogous to the increasing receptive field sizes and tolerance to transformations in biological networks. Note that not all of these operations need to be present in a layer, and it is not always clear what exactly constitutes a layer. Often the last few layers are fully-connected, which is a particular type of convolutional layer with a kernel size of 1×1 px and a feature map usually in the order of several thousands. The output layer is the best example of such fully-connected scheme, where the inputs from the penultimate layer are collapsed to the number of output categories. Thus, in a convnet architecture, an input image is gradually and non-linearly transformed into an output vector that corresponds to the probability of each category known to the model being present in the input.

The resulting architectures have several tens of millions of parameters that need to be learned by the model in order to perform recognition tasks. In a nutshell, first the model is initialized with random weights. An input image is passed through the network, and the resulting category probabilities are compared to the correct label provided by the experimenter. Since the goal is to maximize the chance of a correct response, all incorrect responses count towards the loss of the model, and the weights in the model are updated so as to minimize this loss. As the update happens from top (output) to bottom (input), this procedure is referred to as backpropagation.

In our experiments, all models have been trained on the typical single object categorization task using the ImageNet Large-Scale Visual Recognition Challenge (ILSVRC) 2012 dataset containing about 1.2 million images, divided into 1000 categories [66]. The particular implementation of these models is provided by the Caffe library [67]. We used three models that varied in their architecture and depth:

- **CaffeNet**, a canonical (“vanilla”) implementation of convolutional neural networks offered by the Caffe library that closely resembles the original architecture used by [7] in the ILSVRC-2012. The model consists of five convolutional and three fully-connected layers. The particular model available from Caffe achieves 80.1% top-5 accuracy on the validation set [68].
- **VGG-19 (referred to as VGG-CNN-D in Caffe;** [69]), a runner-up at the ILSVRC 2014 that provides a clean and a very deep implementation of 19 layers. The whole network is analogous to the CaffeNet, but each convolutional layer is split into several convolutional layers (i.e., first and second layers are each split into two, the rest into four) of fixed kernel size (3×3 px) and increasing number of feature maps (64, 128, 256, 512, 512). The particular implementation available from Caffe achieves 88.5% top-5 accuracy on the validation dataset [68].
- **GoogLeNet** [9], a reimplementation of the winning architecture of the ILSVRC 2014 [51], available as part of the Caffe library and trained similarly to CaffeNet. Unlike previous models, this model is composed of the so-called inception modules that compute several convolutions and a max pooling in parallel using different kernel sizes, resulting in a complex non-sequential architecture, which is the reason behind the “jittery” look in our plots. Inception modules have been argued to allow for an increase in available units with little computational cost and to provide access to multiscale representations at each layer [9]. The model has 22 layers that have parameters, thus constituting a very deep model. Given the depth and complexity of the model, there are three auxiliary classifiers spread at mid- and higher-levels of the architecture that aid in training the entire network. The particular model available from Caffe achieves 89.0% top-5 accuracy on the validation set [68].

In our experiments, the final fully-connected layer with 1000 units was reported for all models. In the plots that show all layers, outputs of convolutional and fully-connected layers were reported (thus, no ReLU or pooling layers were reported).

### Correlational analyses

We computed correlations between a particular layer of a model and behavioral data by first computing a dissimilarity matrix between stimuli and then correlating the resulting dissimilarity matrices. We also used a one minus a Pearson correlation as a distance between stimuli as a metric. Since these dissimilarity matrices are symmetric, we used only the upper triangle to compute correlations. Both correlations were computed using the following formula:

$$\frac{\sum_{i=1}^{n}{(x}_{i}-\overset{-}{x}){(y}_{i}-\overset{-}{y})}{\sqrt{\sum_{i=1}^{n}{{(x}_{i}-\overset{-}{x})}^{2}\sum_{i=1}^{n}{{(y}_{i}-\overset{-}{y})}^{2}}},$$

where *x* and *y* correspond to either the outputs of a given model to two different stimuli (for the dissimilarity matrix computation) or the values in the upper triangle of these dissimilarity matrices (for correlational analyses). We also conducted the analyses using a normalized Euclidean distance as a metric for producing dissimilarity matrices, but pattern of results remained the same, indicating that the choice of a metric has little effect on our findings.

The upper and lower bounds of the ceiling performance (shown as a gray band in figures) were estimated using the procedure described in [70]. The upper bound was estimated by computing the average Pearson correlation between each participant’s dissimilarity matrix and the average across all participants after z-scoring data. The lower bound was estimated by computing the average Pearson correlation between each participant’s dissimilarity matrix and the average across the remaining participants after z-scoring data.

### Consistency analyses

In Exp. 1, the consistency between human and model (or between two models) was computed as one minus a normalized squared Euclidean distance between the corresponding accuracy vectors *x* and *y*:

$$1-\frac{\sum_{i=1}^{n}{{(x}_{i}-y_{i})}^{2}}{n}.$$

This metric is a version of the Matching distance that is used for estimating dissimilarity in binary data generalized to the non-logistic case. We chose this metric due to the largely (but not fully) logistic nature of our data (which was not the case in correlational analyses). In particular, this consistency measure is high when most of the responses match, whereas a correlation is very low as there is little variance in the data (i.e., mostly 1’s). The same consistency measure was used for estimating upper and lower bounds of the ceiling performance based on human accuracy instead of the Pearson correlation as described in the correlational analyses.

### Bootstrapping

In order to estimate the reliability of effects in our experiments, we used bootstrapping approach (number of iterations was always 1000). In Fig 1C and Fig 4, we computed 95% confidence intervals by resampling with replacement model responses (that is, the vector of 0’s and 1’s that indicates whether model’s response was correct or not), and computing the mean accuracy at each iteration. The 95% confidence interval was the reported as the 2.5% and 97.5% percentiles of the bootstrapped accuracy distribution

To estimate the reliability of correlations in the correlational analyses (all other figures), we computed bootstrapped confidence intervals as described in [6]. In particular, dissimilarity matrices were resampled with replacement, and the resulting matrices where correlated for a total of 1000 iterations. Note that diagonal entries in the original dissimilarity matrix were undefined, so these entries were removed from the resampled matrices as well. Again, as done in [70], only the upper triangle was used for correlations. In Exp. 2b and Exp. 4, the bootstrapping procedure was carried out in a stratified manner, such that a resampling was done only within each class of stimuli, as done in [6]. In particular, for shape, stimuli were resampled from the same shape class (e.g., a new sample for elongated vertical shapes was sampled from the nine available elongated vertical shapes only), whereas for semantical category, stimuli were resampled from the same category (e.g., a new sample for fruits category was sampled from the six available fruit images only). Resampling without stratification yielded qualitatively comparable results. In Exp. 2a and Exp. 3, no stratification was used because there were too few stimuli (Exp. 2a) or no categories (Exp. 3). Confidence intervals were computed as the 2.5% and 97.5% percentiles of the bootstrap distribution.

To estimate whether shallow, HMAX, and deep models differed, we used a bootstrapped paired-samples significance test (independent-samples significance test gave largely similar results). For each bootstrap resampling, model performance (correlation with the behavioral or pixelwise dissimilarity matrices) was averaged across models within a group, and the difference in average performance was computed for each pair of groups (3 pairwise comparisons in total). Across iterations, this yielded many such differences, which together formed the distribution used for statistical inference (one for each pair of groups). The percentile of scores below zero in each such distribution of differences was reported as the *p*-value.
